# Risk of ischaemic and non-ischaemic heart failure in patients with systemic sclerosis: a population-based study

**DOI:** 10.1093/rheumatology/keaf422

**Published:** 2025-08-06

**Authors:** Majd Bairkdar, Jonas Faxén, Elizabeth V Arkema, Daniel C Andersson, Marie Holmqvist

**Affiliations:** Clinical Epidemiology Division, Department of Medicine Solna, Karolinska Institutet, Stockholm, Sweden; Department of Physiology and Pharmacology, Karolinska Institutet, Stockholm, Sweden; Department of Cardiology, Heart, Vassel and Neuro Theme, Karolinska University Hospital, Stockholm, Sweden; Clinical Epidemiology Division, Department of Medicine Solna, Karolinska Institutet, Stockholm, Sweden; Department of Physiology and Pharmacology, Karolinska Institutet, Stockholm, Sweden; Department of Cardiology, Heart, Vassel and Neuro Theme, Karolinska University Hospital, Stockholm, Sweden; Clinical Epidemiology Division, Department of Medicine Solna, Karolinska Institutet, Stockholm, Sweden; Medical Unit Gastroenterology, Dermatolgy and Rheumatology, Karolinska University Hospital, Stockholm, Sweden

**Keywords:** systemic sclerosis, heart failure, ischaemic heart disease

## Abstract

**Objectives:**

To investigate the risk of incident ischaemic and non-ischaemic heart failure (HF) in a population-based cohort of all patients with SSc in Sweden compared with the general population.

**Methods:**

We identified patients with incident SSc 2004–19 using nationwide Swedish registers and age- and sex-matched comparators from the general population (1:10). We started follow-up from the date of SSc diagnosis, the same date was assigned to the respective comparators. Our primary outcome was incident HF [International Statistical Classification of Diseases and Related Health Problems (ICD)-10: I50] as main diagnosis stratified into ischaemic and non-ischaemic HF depending on whether a visit listing ischaemic heart disease (ICD-10: I20–I25) was recorded before HF diagnosis or not. We used flexible parametric models to estimate hazard ratios (HRs) over time since SSc diagnosis.

**Results:**

The study cohort comprised 1598 patients with SSc and 16 616 comparators. During follow-up, 101 (6%) patients with SSc developed HF compared with 378 (2%) of the comparators. Ischaemic HF represented 35% and non-ischaemic HF represented 65% of all HF cases in both groups. The relative risk of HF overall, ischaemic HF and non-ischaemic HF was highest soon after SSc diagnosis. HR at the end of the first year of follow-up was 5.7 (95% CI 4.2–7.9) for HF overall, 6.7 (95% CI 3.5–13.0) for ischaemic HF and 5.8 (95% CI 4.0–8.3) for non-ischaemic HF.

**Conclusion:**

SSc is associated with both ischaemic and non-ischaemic HF, emphasizing the role of other mechanisms than ischaemia, such as myocyte dysfunction, myocardial fibrosis and microvascular impairment, in the development of HF in SSc.

## Graphical Abstract

**Figure:**
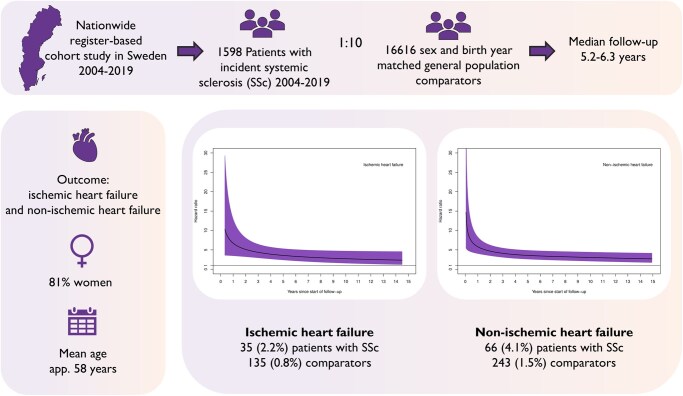

Rheumatology key messages

- SSc is associated with both ischaemic and non-ischaemic heart failure.
- Mechanisms other than ischaemia are likely implicated, potentially myocardial fibrosis and microvascular dysfunction.
- Screening for heart failure in SSc is crucial, even without traditional cardiovascular risk factors.

## Introduction

SSc is a rare autoimmune disease characterized by autoimmunity, endothelial dysfunction and progressive fibrosis [1]. SSc has a heterogeneous presentation that can involve the skin, lungs, kidneys and the cardiovascular system [1], with cardiac involvement being one of the leading causes of death [2]. Cardiac involvement comprises a wide range of manifestations such as myocardial infarction, arrhythmia and heart failure (HF) [3]. In a previous study, cardiac involvement was present in 44% of patients with SSc; 26% of them had HF [4]. It is consistently reported that SSc is associated with a higher risk of developing HF compared with individuals without SSc [5–7], and it has been suggested that factors related to SSc itself could be involved in HF development [8]. However, there has been insufficient investigation into what is the primary driver for HF in SSc. It could be that HF in SSc is a manifestation secondary to SSc but it could also be a consequence of the known increased risk of myocardial infarction [6, 9, 10], one of the major risk factors for HF in the general population [11, 12].

In this study, we therefore aimed to investigate the risk of ischaemic and non-ischaemic HF in a large population-based cohort of patients with SSc compared with the general population. We also aimed to explore the temporal trend of HF onset in relation to age and to SSc diagnosis, information of high clinical value for both patients with SSc and their healthcare providers.

## Methods

### Setting and study design

This is a population-based matched cohort study using nationwide Swedish registers. In Sweden, healthcare is tax-funded and equally accessible to all residing in Sweden. Data in these registers are gathered regardless of disease status and socioeconomic status, making it possible to conduct high quality and generalizable nationwide studies of rare diseases [13]. Patients with SSc are usually cared for by rheumatologists in an outpatient setting.

### Data sources

Data on all hospitalization and data on visits in the outpatient specialized, non-primary, care in Sweden are captured in the National Patient Register (NPR) with almost complete coverage since 1987 for inpatient care and 2001 for outpatient care. A main diagnosis and up to 30 contributory diagnoses are registered for every hospitalization and visit according to the International Statistical Classification of Diseases and Related Health Problems (ICD) codes [14]. Demographic data such as birth date, sex and migration are captured in the Total Population Register (TPR) [15]. The Prescribed Drug Register (PDR) collects data on all dispensed drugs from pharmacies in Sweden since 1 July 2005 such as ATC code (Anatomical Therapeutic Chemical Classification System), in addition to dosage form, package size, date of dispensation, etc. [16]. Data on education for all adults in Sweden are collected annually since 1990 in the Longitudinal Integrated Database for Health Insurance and Labour Market Studies [17].

### Study population

We identified all patients with incident SSc in Sweden between 2004 and 2019 from the NPR. We defined incident SSc as the following: at least two visits or hospitalizations where SSc (ICD-10: M34.0, M34.1, M34.8, M34.9) was coded as the main diagnosis, the first ever visit occurred between 1 January 2004 and 31 December 2019 and the second visit after no more than 1 year from the first, and at least one of those two visits had to be made in either an internal medicine or rheumatology department. We considered only those who were ≥18 years at the date of the second visit. We identified comparators from the general population matched to SSc patients 10:1 on sex and birth year using the TPR. We excluded individuals who had any visit or hospitalization where HF (ICD-10: I50) was coded as main or contributory diagnosis before the second visit, to include only individuals free from HF at start of follow-up.

### Outcome

Our outcome was incident HF stratified into ischaemic and non-ischaemic HF. We defined HF diagnosis as either (i) any visit after start of follow-up coded ICD-10: I50 as a main diagnosis from the outpatient specialized care or (ii) any hospitalization after start of follow-up with discharge code ICD-10: I50 as a main diagnosis from inpatient care. The validity of HF diagnosis in the outpatient specialized care has not been studied in Sweden, but its validity as main diagnosis in inpatient care is high; positive predictive value >94% for definite or probable diagnosis [18, 19]. We classified HF events with a prior visit listing ischaemic heart disease (IHD) (ICD-10: I20–I25) as a main or contributory diagnosis as ischaemic HF. We classified those without prior IHD as non-ischaemic HF.

### Follow-up

Start of follow-up was the date of the second SSc visit, and the same date was assigned to the matched comparators. In the analysis of HF overall, we followed participants until they developed HF overall or were censored due to death, emigration or study end (31 December 2019), whichever occurred first. In the analysis of ischaemic HF, we followed participants until they developed ischaemic HF or were censored in case of non-ischaemic HF, death, emigration or study end (31 December 2019). In the analysis of non-ischaemic HF, we excluded those with a history of IHD before start of follow-up and followed the remaining participants until they developed non-ischaemic HF, or were censored due to IHD, death, emigration or study end (31 December 2019).

### Other covariates

We considered the following covariates: age at start of follow-up (continuous variable), sex (women *vs* men) and education level at start of follow-up (classified into ≤9 years, 10–12 years and >12 years). We also identified the following comorbidities from the NPR and PDR prior to start follow-up: atrial fibrillation and flutter, renal diseases, asthma/chronic obstructive pulmonary disease, diabetes mellitus, myocarditis, pericarditis, cardiomyopathy, hyperlipidaemia, ischaemic stroke, peripheral artery disease and hypertension. We provide the definitions of these comorbidities in Supplementary data S1.

### Ethical approval

This study was approved by the Swedish Ethical Review Authority, number 2017–2000/31 and 2020–04529.

### Statistical methods

We described the characteristics of the study population at start of follow-up, after excluding all with a prior diagnosis of HF, using means with s.d., medians with interquartile range (IQR) and percentages as deemed appropriate. We compared the occurrence of HF before start of follow-up in the two groups using χ^2^ test.

We used time since start of follow-up as time scale. We estimated crude incidence rates of HF overall, ischaemic HF and non-ischaemic HF separately in patients with SSc and their comparators. To study the relative risk of each outcome over time, we used the flexible parametric models to estimate age- and sex-adjusted time-varying hazard ratios (HRs) by allowing for SSc to have a time-dependent effect [20].

To explore the effect of known, potential and measured confounders, we estimated HRs of each outcome adjusting for age, sex, education level and the above-mentioned comorbidities at start of follow-up, except myocarditis, pericarditis and cardiomyopathy due to few cases. We also adjusted the model assessing HF overall for history of IHD. In an additional analysis, we estimated the relative risk of HF overall using age as time scale to explore how the relative risk develops over increasing age.

We conducted several sensitivity analyses to assess the robustness of our results. First, we started follow-up 90 days after the second SSc visit to ensure only truly incident cases of HF were included in the analysis. Prevalent previously undetected cases that might be incidentally detected during the screening process patients usually undergo at SSc diagnosis should not be captured in this analysis. Second, we redefined HF as main or contributory diagnosis from the outpatient or the inpatient care. Third, we excluded those with cardiomyopathy prior to start of follow-up. To further explore the role of SSc itself in the risk of developing HF, we excluded all individuals with any of the comorbidities mentioned above and then estimated the relative risk of HF overall. In addition, we considered in the analysis of ischaemic HF only those who had an IHD event after start of follow-up and excluded those with IHD before start of follow-up. Some patients with newly detected HF may undergo coronary angiography as part of the clinical work up after a newly diagnosed HF and as a consequence of the angiography be diagnosed with IHD after the diagnosis of HF itself, i.e. the ICD code indicating HF may precede the ICD code indicating IHD. Therefore, to further test the robustness of our definition of ischaemic and non-ischaemic HF, we defined ischaemic HF as an IHD coded visit up to 30 days after HF diagnosis and non-ischaemic HF as no IHD coded visit up to 30 days after HF diagnosis. Finally, since IHD and non-ischaemic HF might be considered as competing risks in the analysis of non-ischaemic HF and ischaemic HF, respectively, we estimated the relative risk using a Fine and Gray competing risks regression model [21].

We conducted the analyses incorporating the above-mentioned comorbidities only on participants with start of follow-up starting from 1 January 2006, since the PDR has complete coverage since 1 July 2005, in order to allow for a period of at least 6 months to identify drugs of interest before start of follow-up.

We performed the statistical analyses using R version 4.3.2 (R Foundation for Statistical Computing, Vienna, Austria) [22].

## Results

There were 1722 patients with incident SSc included between 2004 and 2019 and 16 983 comparators from the general population. A larger proportion of patients with SSc had HF prior to start of follow-up than the comparators (*P*-value <0.0001); 124 (7%) patients with SSc and 367 (2%) comparators. More than 50% of those patients with SSc received their first ever code for HF within 1 year prior to start of follow-up, compared with only 18% of the comparators (Supplementary Fig. S1).

After excluding participants with HF before start of follow-up, the remaining 1598 patients with SSc and 16 616 comparators comprised our study population. Characteristics of the participants at start of follow-up are presented in Table 1. The mean age at start of follow-up was 57.7 (s.d. 14.7) years and 58.3 (s.d. 14.8) years in patients with SSc and the comparators, respectively. In both groups, 81% of the participants were women.

**Table 1.** Characteristics at start of follow-up

	Patients with SSc (*n* = 1598)	General population comparators (*n* = 16 616)
Age at start of follow-up, years, mean (s.d.)	57.7 (14.7)	58.3 (14.8)
Sex, *n* (%)		
Women	1296 (81)	13 439 (81)
Men	302 (19)	3177 (19)
Highest attained education, *n* (%)		
≤9 years	358 (22)	3881 (23)
10–12 years	688 (43)	6990 (42)
>12 years	508 (32)	5424 (33)
Missing	44 (3)	321 (2)
Comorbidities at start of follow-up^a^, *n* (%)		
Diabetes	83 (6)	1042 (7)
Atrial fibrillation and flutter	56 (4)	415 (3)
Ischaemic heart disease	86 (6)	683 (5)
Renal diseases	57 (4)	369 (3)
Asthma/COPD	337 (24)	2792 (19)
Hypertension	526 (37)	3891 (26)
Myocarditis	<5	12 (0.1)
Pericarditis	16 (1)	25 (0.2)
Cardiomyopathy	5 (0.4)	22 (0.1)
Ischaemic stroke	31 (2)	321 (2)
Peripheral artery disease	49 (3)	118 (1)
Hyperlipidaemia	237 (17)	2632 (18)

Characteristics at start of follow-up of patients with incident SSc 2004–19 and general population comparators with no prior heart failure diagnosis. Percentages in parentheses represent the proportion of individuals exhibiting the characteristic within the respective category.

a Comorbidities evaluated only in participants with start of follow-up from 1 January 2006 (1421 patients with SSc and 14 809 general population comparators). COPD: chronic obstructive pulmonary disease.

### Heart failure overall

**Figure 1.**
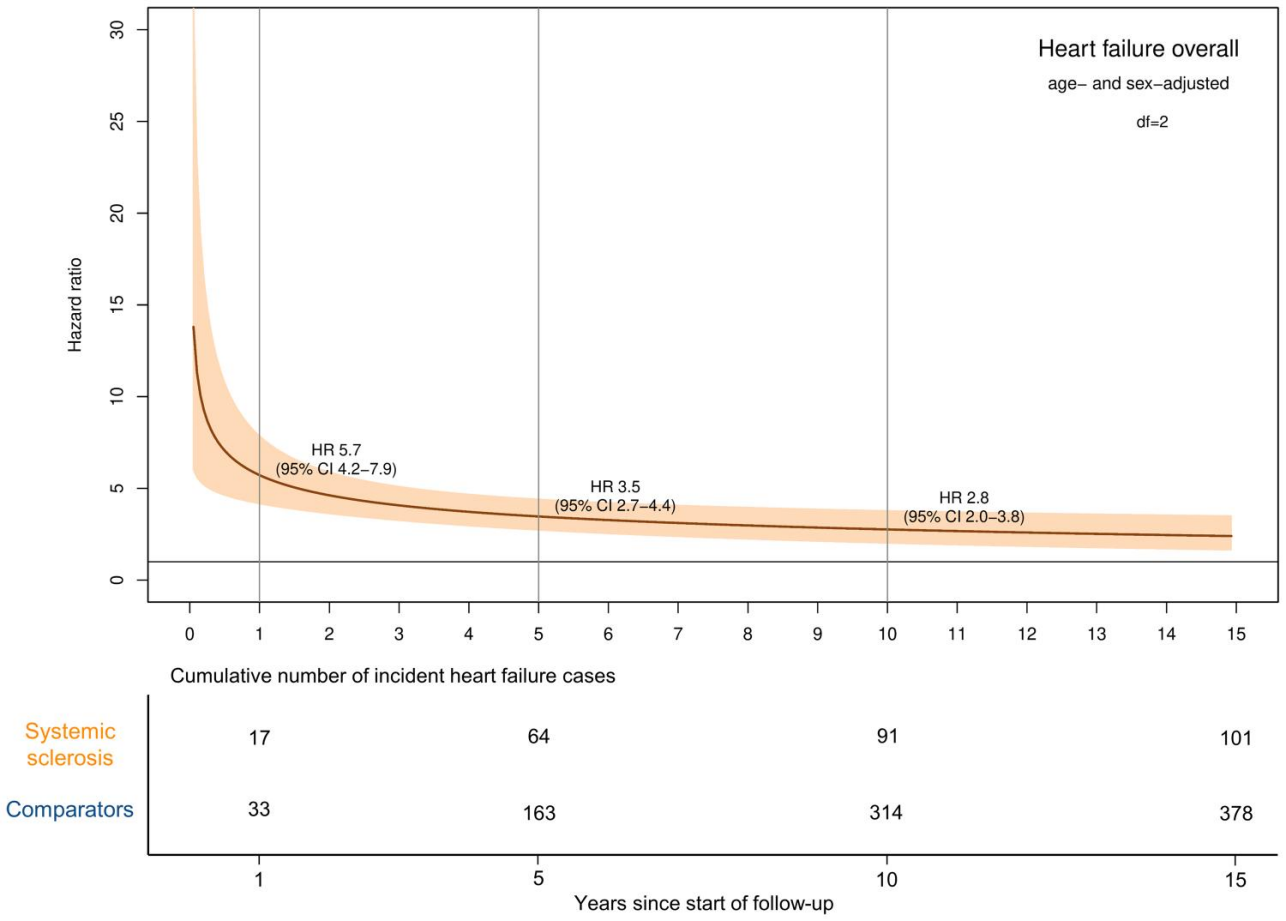
Hazard ratio of heart failure overall in patients with incident SSc 2004–19 compared with the general population comparators using flexible parametric models adjusted for age and sex, allowing for SSc to have a time-dependent effect

The median follow-up was 5.2 years (IQR 6.7) in patients with SSc and 6.3 years (IQR 7.3) in the comparators. During follow-up, 101 (6%) patients with SSc and 378 (2%) comparators developed HF (Supplementary Fig. S2). The crude incidence rates were 103.3 (95% CI 84.1–125.5) and 32.9 (95% CI 29.7–36.4) per 10 000 person-years, respectively (Table 2). The estimated age- and sex-adjusted HR of HF in patients with SSc was highest directly after start of follow-up and then declined gradually but was significant during the entire follow-up period; HR was 5.7 (95% CI 4.2–7.9) at the end of the first year of follow-up, 3.5 (95% CI 2.7–4.4) at the end of the fifth year and 2.8 (95% CI 2.0–3.8) at the end of the tenth year (Fig. 1). We obtained similar estimates in the fully adjusted model (Supplementary Fig. S3).

**Table 2.** Incidence rates of heart failure

	Patients with SSc			General population comparators			
	*N*	Person-years	IR with 95% CI^a^	*N*	Person-years	IR with 95% CI^a^	Rate difference with 95% CI
Heart failure overall	101	9779	103.3 (84.1–125.5)	378	114 759	32.9 (29.7–36.4)	70.3 (49.9–90.8)
Women	68	7945	85.6 (66.5–108.5)	257	91 817	28.0 (24.7–31.6)	57.6 (37.0–78.2)
Men	33	1835	179.9 (123.8–252.6)	121	22 942	52.7 (43.8–63.0)	127.1 (65.0–189.2)
Ischaemic HF	35	9779	35.8 (24.9–49.8)	135	114 759	11.8 (9.9–13.9)	24.0 (12.0–36.0)
Non-ischaemic HF	66	8974	73.5 (56.9–93.6)	243	107 671	22.6 (19.8–25.6)	51.0 (33.0–68.9)

Crude incidence rate and rate difference of heart failure overall, stratified by sex and stratified into ischaemic and non-ischaemic heart failure in patients with incident SSc 2004–19 and general population comparators.

a IRs are incidence rates per 10 000 person-years. HF: heart failure.

**Figure 2.**
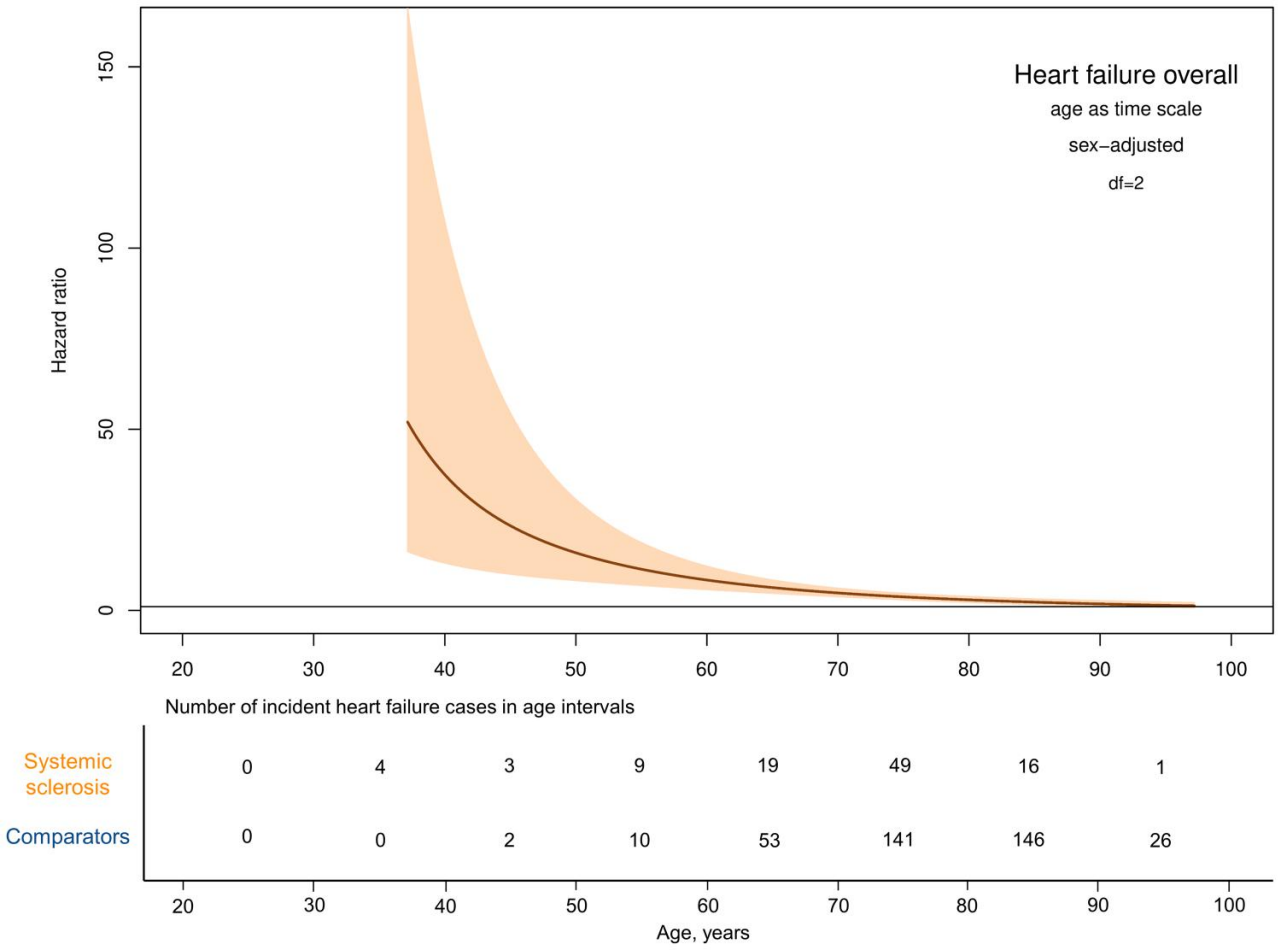
Hazard ratio of heart failure overall in patients with incident SSc 2004–19 compared with the general population comparators using increasing age as time scale using flexible parametric models adjusted for sex, allowing for SSc to have a time-dependent effect

Using age as time scale, the relative risk of developing HF was significantly higher in patients with SSc than the comparators, particularly until the age of 60 (Fig. 2). It is worth mentioning that four patients with SSc developed HF in the interval 30–40 years, but none of the comparators did.

### Ischaemic heart failure

**Figure 3.**
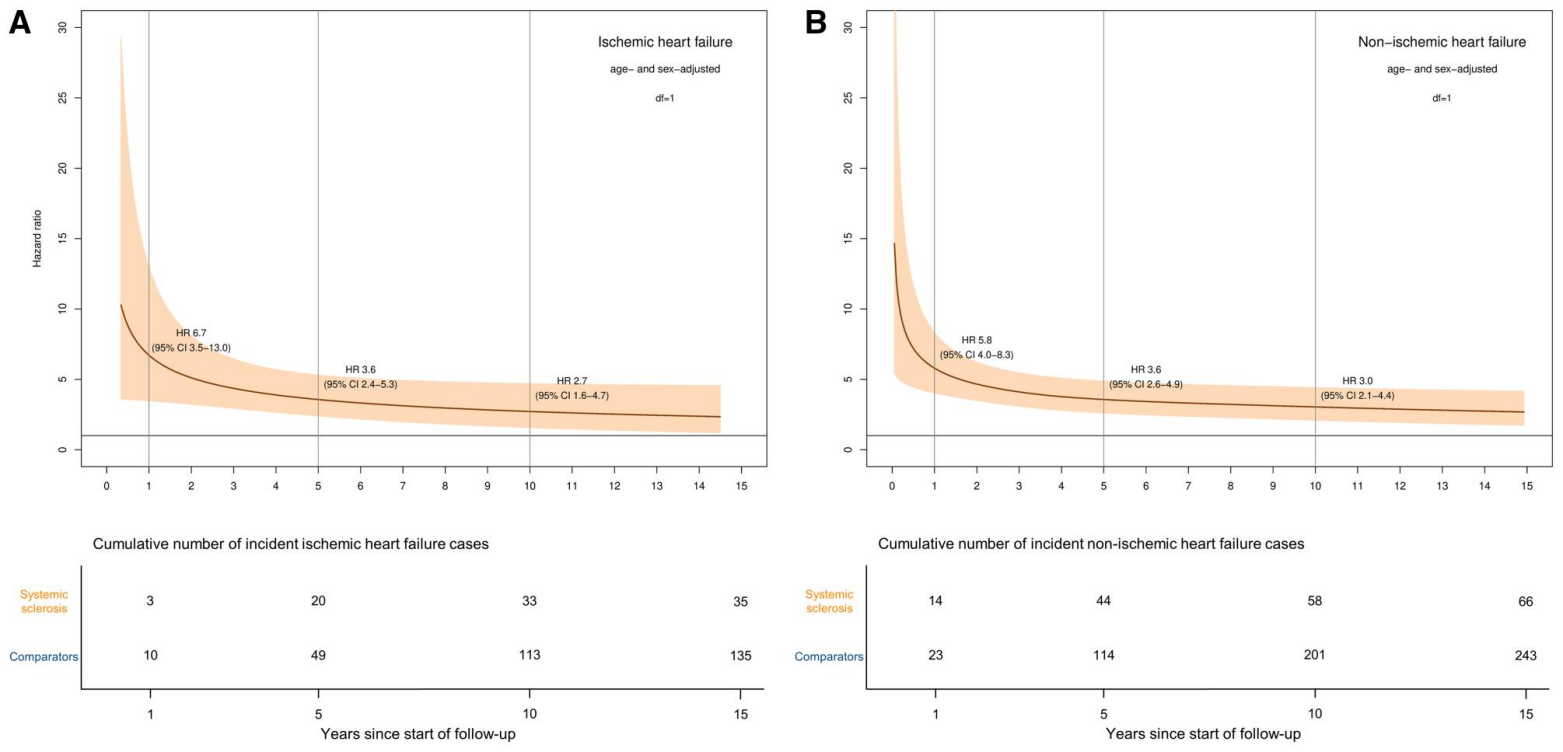
Hazard ratio of ischaemic heart failure (**A**) and non-ischaemic heart failure (**B**) in patients with incident SSc 2004–19 compared with the general population comparators using flexible parametric models adjusted for age and sex, allowing for SSc to have a time-dependent effect

During follow-up, 35 patients with SSc and 135 comparators developed ischaemic HF (Supplementary Fig. S4). The incidence rate was 35.8 (95% CI 24.9–49.8) per 10 000 person-years in patients with SSc and 11.8 (95% CI 9.9–13.9) in the comparators (Table 2). Patients with SSc were younger at ischaemic HF diagnosis, mean age 74 *vs* 79 years. Similar to HF overall, the relative risk was highest early during follow-up and then decreased, both in the age- and sex-adjusted model and the fully adjusted model (Fig. 3A and Supplementary Fig. S5).

### Non-ischaemic heart failure

Non-ischaemic HF occurred in 66 patients with SSc and 243 comparators (Supplementary Fig. S6) with an incidence rate of 73.5 (95% CI 56.9–93.6) and 22.6 (95% CI 19.8–25.6) per 10 000 person-years, respectively (Table 2). Mean age at non-ischaemic HF diagnosis was 69 years in patients with SSc and 78 years in the comparators. We observed a similar trend of HR being highest early in the follow-up period and then declining slowly in both models (Fig. 3B and Supplementary Fig. S7).

### Sensitivity analyses

Starting follow-up 90 days after the second SSc visit did not alter our results (Supplementary Table S1 and Figs S8 and S9). The incidence rates of HF as main or contributory diagnosis and when excluding those with a history of cardiomyopathy are presented in Supplementary Table S1. Consistent with the main analysis, the incidence rate of HF in patients with SSc was ∼3 times higher than that of the comparators. In the analysis where we excluded individuals with any of the comorbidities mentioned above, the higher risk of HF in patients with SSc compared with the matched comparators became more prominent, especially early during the follow-up period (Supplementary Table S1 and Fig. S10). Likewise, in the analysis excluding those with IHD prior to start of follow-up, the increased relative risk of ischaemic HF was more pronounced (Supplementary Fig. S11). Our results also remained unchanged when we allowed for IHD up to 30 days after start of follow-up to be classified as ischaemic HF (Supplementary Table S2). Finally, using competing risks regression model, the age- and sex-adjusted HR was 3.6 (95% CI 2.5–5.3) for ischaemic HF and was 3.8 (95% CI 2.9–5.0) for non-ischaemic HF in patients with SSc compared with the comparators.

## Discussion

This population-based study is, to the best of our knowledge, the first to investigate the risk of ischaemic and non-ischaemic HF in patients with SSc. We confirmed that patients with SSc are at increased risk of developing HF and provide further evidence that this is true for individuals both with and without a history of IHD, and in individuals without a history of cardiovascular diseases or other related comorbidities. Our results were robust across several sensitivity analyses.

Previous studies have reported an association between SSc and HF. In Taiwan, patients with SSc had over 3-fold increased risk of developing HF compared with matched individuals [5]. A US cohort study found a similar relative risk of HF in patients with incident SSc (HR of 3.6) [6]. A Danish study found that SSc was associated with higher risk of HF in both women and men and in patients younger and older than 55 years at SSc diagnosis [23]. In line with those studies, the risk of HF overall in our study was significantly higher in patients with SSc than the comparators. Patients with SSc showed highest relative risk of developing HF within the first few years after start of follow-up compared with the matched comparators (HR was 5.7 at the end of the first year of follow-up and 3.5 at the end of the fifth year). These findings remained consistent after adjusting for cardiovascular and other related comorbidities.

An extensive screening is routinely conducted when SSc diagnosis is made to evaluate potential organ involvement including echocardiography, primarily to screen for pulmonary arterial hypertension, a known cardiovascular manifestation in SSc. To avoid misclassifying prevalent previously undetected HF cases as incident cases that were incidentally detected during the screening process, we conducted a sensitivity analysis where we started follow-up 90 days later. The relative risk of HF remained almost unchanged. An important point to consider here is that we defined HF as a main diagnosis from the inpatient care or the outpatient specialized care. HF is coded as main diagnosis almost exclusively in internal medicine, cardiology or in some cases geriatrics departments. This means that using our definition we most likely identified only those with clinically manifest HF since they were either hospitalized or treated at specialized outpatient clinics due to HF. HF detected incidentally during the screening process would most likely not lead to a hospitalization or a visit in the specialized care with HF as main diagnosis.

In our study, we also demonstrated that SSc was not only associated with higher risk of HF but also a substantially higher risk at younger age compared with their counterparts from the comparator group (Fig. 2). We also observed that SSc was associated with higher risk of HF even in individuals without cardiovascular and other related comorbidities, stressing the role of SSc itself in HF.

In agreement with a study from Denmark [7], we found that the proportion of patients with SSc who had HF before start of follow-up was higher than their comparators. This observation can be attributed, at least partly, to diagnostic delay in addition to the rather vague and multi-faceted debut of SSc leading to some patients probably being diagnosed with SSc-related manifestations, including cardiovascular diseases, prior to SSc diagnosis itself. The fact that more than half of patients with SSc excluded due to HF before start of follow-up received their first ever code for HF within 1 year prior to start of follow-up supports this explanation. We therefore expect the true incidence rate of HF in patients with SSc to be higher than our estimate.

The association between SSc and increased risk of myocardial infarction is well-established [6, 9, 10]. We also know that myocardial infarction is a major risk factor for HF in the general population [11, 12]. It has been suggested however that cardiac involvement in SSc, including HF, is partly the result of microvascular pathology leading to perfusion–reperfusion injury in addition to myocardial fibrosis [8]. Chronic small vessel vasculopathy in addition to structural alterations such as diastolic dysfunction has also been described [24, 25]. Cardiac magnetic resonance (CMR) imaging has showed signs of microvascular dysfunction and focal and diffuse fibrosis of the myocardium in asymptomatic patients with SSc [26]. Moreover, systemic inflammatory stress in SSc can lead to impaired function of multiple cell types within the myocardium including cardiomyocytes. Indeed, reduced myofibrillar contractile function has been showed in cardiomyocytes from ventricular biopsies in SSc patients [27]. To explore how much of the HF risk could be attributed to SSc or IHD, we stratified HF into ischaemic and non-ischaemic depending on whether IHD occurred prior to HF or not. The relative risk of both subtypes was significantly higher in patients with SSc than in the comparators with similar temporal pattern to HF overall; we observed the highest risk during the first few years after start of follow-up. Since the association between SSc and myocardial infarction which represents a major part of IHD, is demonstrated in the literature [6, 9, 10], it was expected that we observe an increased risk of ischaemic HF in patients with SSc. However, we found that ischaemic HF was not the dominating subtype of HF in this patient group. On the contrary, the proportions of ischaemic and non-ischaemic HF of all HF cases were comparable in patients with SSc and their comparators. This finding indicates that SSc is associated with HF through additional mechanisms other than ischaemia, a finding that stresses the role of SSc-related manifestations, for example myocardial fibrosis and microvasculature dysfunction, in the occurrence of HF in SSc. CMR has a significant role in detecting fibrotic and inflammatory changes, beyond changes detected by echocardiography, in the myocardium of patients with SSc [28]. T1 mapping CMR is a novel method to detect diffuse myocardial fibrosis in SSc, more sensitive than standard CMR and echocardiography [29]. On the other hand, PET is used to noninvasively detect cardiac microvascular dysfunction [30] which has been reported more frequent in patients with SSc, particularly in patients with RP, than patients with other autoimmune diseases and controls with primary RP [31]. The use of these modalities should be tailored to the characteristics and clinical profile of each patient.

Thanks to the nationwide Swedish registers with almost complete coverage, this study comprised a cohort of all patients with SSc in the country. The inclusion of matched individuals from the general population provided population-based background risks contextualizing our findings. The follow-up of up to 16 years allowed for capturing information on HF that develop over a longer time period. In addition, the validity of HF diagnosis in the registers is high [18, 19]. The coverage of IHD is almost complete as patients presenting with IHD are predominantly managed in the specialized care.

However, this study has some limitations. First, we were unable to describe the heterogeneity of SSc using the NPR which contains information on ICD codes but not clinical characteristics. We had no data on autoantibody pattern and disease subset depending on skin involvement; limited, diffuse and sine SSc. Furthermore, echocardiographic parameters such as ejection fraction, diastolic function, pulmonary arterial pressure as well as NT-proBNP levels are not captured in the NPR. We were therefore unable to distinguish between HF with reduced ejection fraction and HF with preserved ejection fraction. Data on coronary angiography were not available to further refine our classification of HF into ischaemic and non-ischaemic. Unmeasured confounding should also be mentioned due to the lack of information on body mass index, physical activity and smoking. In addition, data from primary care are not captured in the NPR which may introduce differential outcome misclassification. Since patients with SSc routinely are followed in an outpatient setting by a rheumatologist, they may be more likely to meet a cardiologist or an internist if they develop heart failure than those without SSc who may be followed mainly by the primary care (care that is not captured by the NPR). However, as mentioned above, we expect the true incidence rate of HF in patients with SSc to be higher than our estimate.

In conclusion, this study confirms that SSc is associated with HF. This study highlights the need to investigate other mechanisms than ischaemia, potentially myocardial fibrosis, impaired myocyte function and microvascular dysfunction, in the aetiology of HF in SSc. Moreover, SSc is associated with HF at younger age than SSc-free matched comparators. It is therefore important to screen for HF, also in patients with SSc without traditional risk factors for HF. Methods useful for the detection of HF such as NT-proBNP level analysis should be conducted routinely in this patient group. Advanced modalities beyond echocardiography such as CMR and PET can be useful in exploring HF aetiology in selected cases.

## Supplementary Material

keaf422_Supplementary_Data

## Data Availability

The data underlying this article cannot be shared outside Karolinska Institutet due to regulatory constraints.
