# Supplementary material for: Risk of ischaemic and non-ischaemic heart failure in patients with systemic sclerosis: a population-based study
Source: Rheumatology (Oxford). 2025 Aug 6;64(12):6184–91. doi: 10.1093/rheumatology/keaf422 (PMC12671861; doi:10.1093/rheumatology/keaf422)
Supplement: keaf422_Supplementary_Data [file keaf422_supplementary_data.docx]

**Risk of ischemic and non-ischemic heart failure in patients with systemic sclerosis:
a population-based study**

Majd Bairkdar^1^, Jonas Faxén^2, 3^, Elizabeth V. Arkema^1^, Daniel C Andersson^2, 3^, Marie Holmqvist^1, 4^

^1^Clinical epidemiology division, Department of medicine Solna, Karolinska Institutet, Stockholm, Sweden

^2^Department of physiology and pharmacology, Karolinska Institutet, Stockholm, Sweden.

^3^Department of cardiology, Heart, Vassel and Neuro Theme, Karolinska University Hospital, Stockholm, Sweden

^4^Medical Unit Gastroenterology, Dermatolgy and Rheumatology, Karolinska University Hospital, Stockholm, Sweden

**Supplementary file**

**Supplementary data S1**. The definitions of comorbidities at start of follow-up.

- Atrial fibrillation and flutter:
  any visit prior to start of follow-up coded (ICD-10: I48) as a main or contributory diagnosis.
- Ischemic heart disease:

any visit coded (ICD-10: I20-I25) as a main or contributory diagnosis (1), prior to incident heart failure diagnosis, regardless of whether it was recorded prior to or after start of follow-up. It was reported that a hospital discharge with ICD-9 code for acute myocardial infarction 410 (corresponding to ICD-10: I21-I22), which represent to a significant part of ischemic heart disease, had a validity of 86% (1).

- Renal diseases:
  any visit prior to start of follow-up coded (ICD-10: N00– N19) as a main or contributory diagnosis.
- Asthma/Chronic obstructive pulmonary disease (Asthma/COPD):
  either 1) any visit prior to start of follow-up coded (ICD-10: J45-J46) or (ICD-10: J44) as a main or contributory diagnosis or 2) at least one dispensed prescription of any asthma/COPD drug (ATC: R03AC, R03AK, R03BA, R03BB) prior to start of follow-up. These ATC codes were found to be able to function as a proxy for asthma/COPD in a Swedish study since they encompass adrenergics, combination drugs, steroids, and anti-cholinergics (2).
- Diabetes mellitus:

either 1) any visit prior to start of follow-up coded (ICD-10: E10-E14) as a main or contributory diagnosis or 2) at least two dispensed prescriptions of any glucose-lowering drug (ATC: A10) prior to start of follow-up. This ATC code was considered to be able to function as a proxy for pharmacologically treated diabetes in Sweden, both type 1 and type 2, since it encompasses both insulin and other subgroups of glucose-lowering drugs (3).

- Myocarditis:

any visit prior to start of follow-up coded (ICD-10: I40-41) as a main or contributory diagnosis.

- Pericarditis:

any visit prior to start of follow-up coded (ICD-10: I30-I32) as a main or contributory diagnosis.

- Cardiomyopathy:

any visit prior to start of follow-up coded (ICD-10: I42) as a main or contributory diagnosis.

- Hyperlipidemia:

either 1) any visit prior to start of follow-up coded (ICD-10: E78) as a main or secondary diagnosis or 2) at least two dispensed prescriptions of lipid modifying agents (ATC: C10) prior to start of follow-up (4).

- Ischemic stroke:

any visit prior to start of follow-up coded (ICD-10: I63-I64) as a main or contributory diagnosis.

- Peripheral artery disease:

any visit prior to start of follow-up coded (ICD-10: I702 and I739) as a main or contributory diagnosis.

- Hypertension:
  this definition is based on Danish data where medical and epidemiological specialists proposed definitions to identify chronic diseases using register data (4):
  either 1) any visit prior to start of follow-up coded (ICD-10: I10-I15) as a main or contributory diagnosis or 2) at least one dispensed prescription of at least two of the following classes of antihypertensive agents prior to start of follow-up:
- α Adrenergic blockers (C02A, C02B, C02C)
- non-loop diuretics (C02DA, C02L, C03A, C03B, C03D, C03E, C03X, C07C, C07D, C08G, C09BA, C09DA, C09XA52)
- vasodilators (C02DB, C02DD, C02DG, C04, C05)
- β blockers (C07)
- calcium channel blockers (C07F, C08, C09BB, C09DB)
- renin-angiotensin system inhibitors (C09)


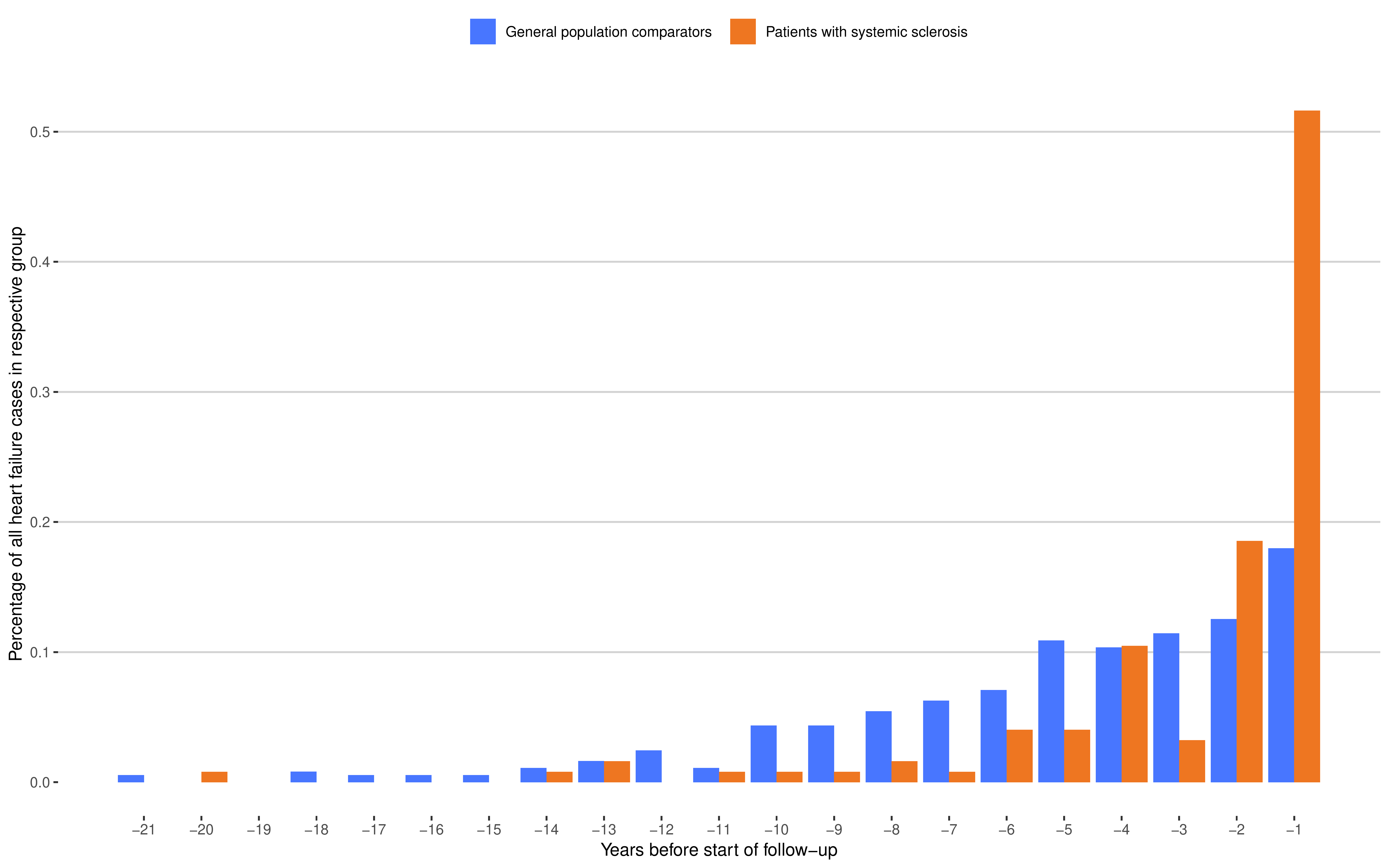


**Supplementary figure S1**. Percentage of all heart failure cases in patients with systemic sclerosis and their comparators, respectively, over years prior to start of follow-up.


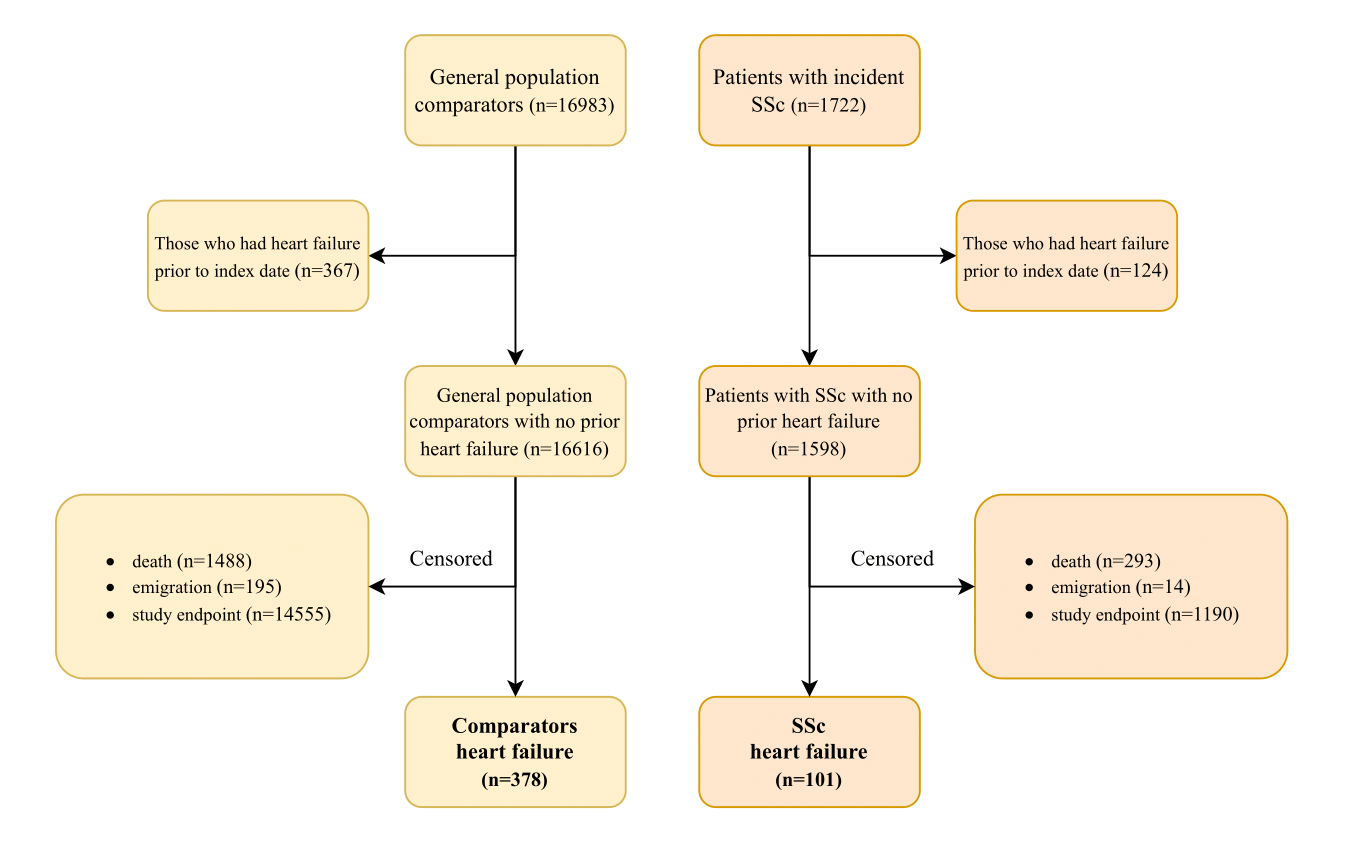


**Supplementary figure S2**. Flowchart of heart failure overall.

**
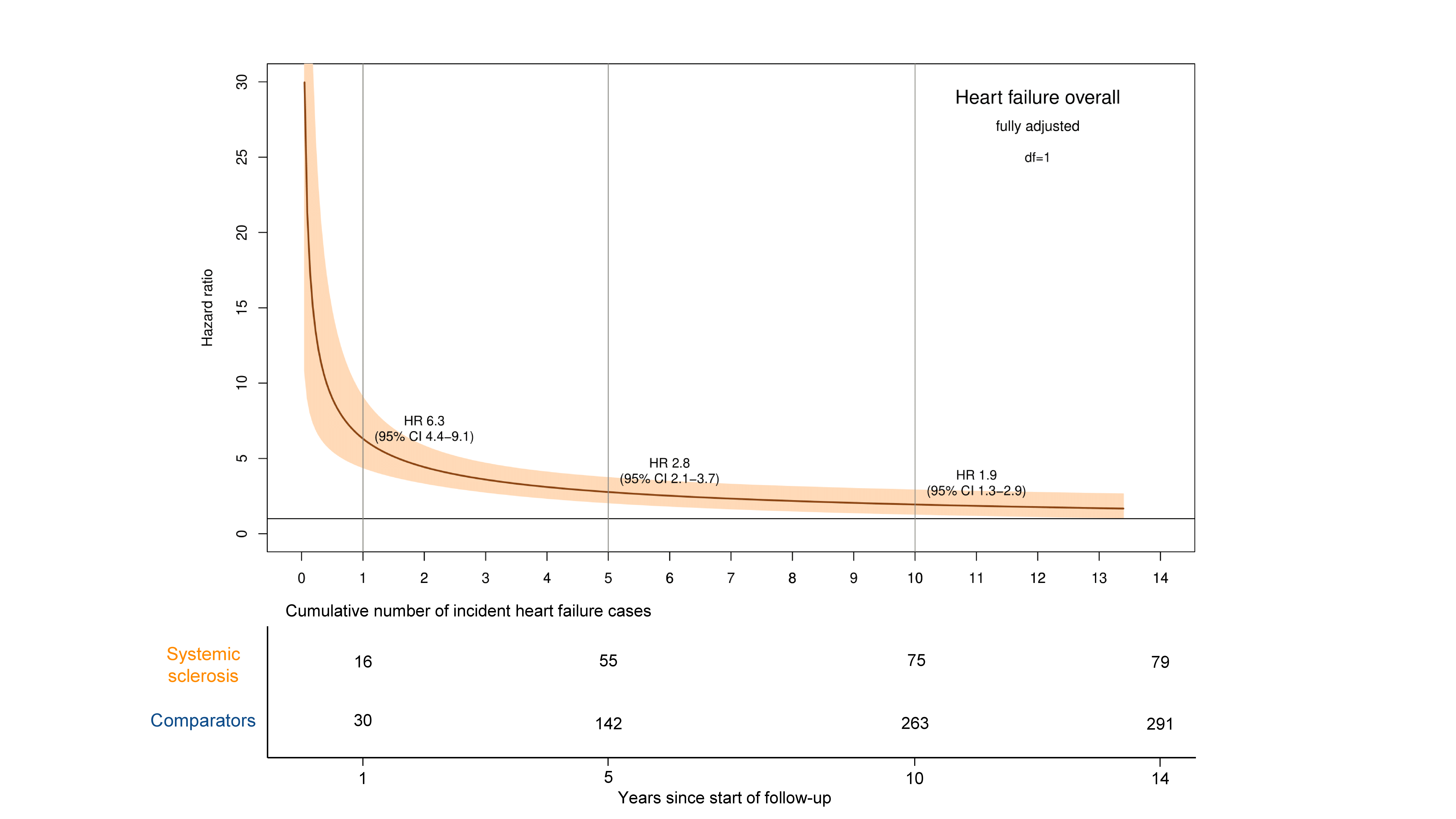
**

**Supplementary figure S3**. Hazard ratio of heart failure overall in patients with systemic sclerosis compared to the general population comparators with **start of follow-up from January 1, 2006 (1,421 patients with systemic sclerosis and 14,809 matched comparators)** using flexible parametric models adjusted for age, sex, education level, ischemic heart disease, atrial fibrillation and flutter, renal diseases, asthma/chronic obstructive pulmonary disease, diabetes mellitus, hyperlipidaemia, ischemic stroke, peripheral artery disease, and hypertension, allowing for systemic sclerosis to have a time-dependent effect.


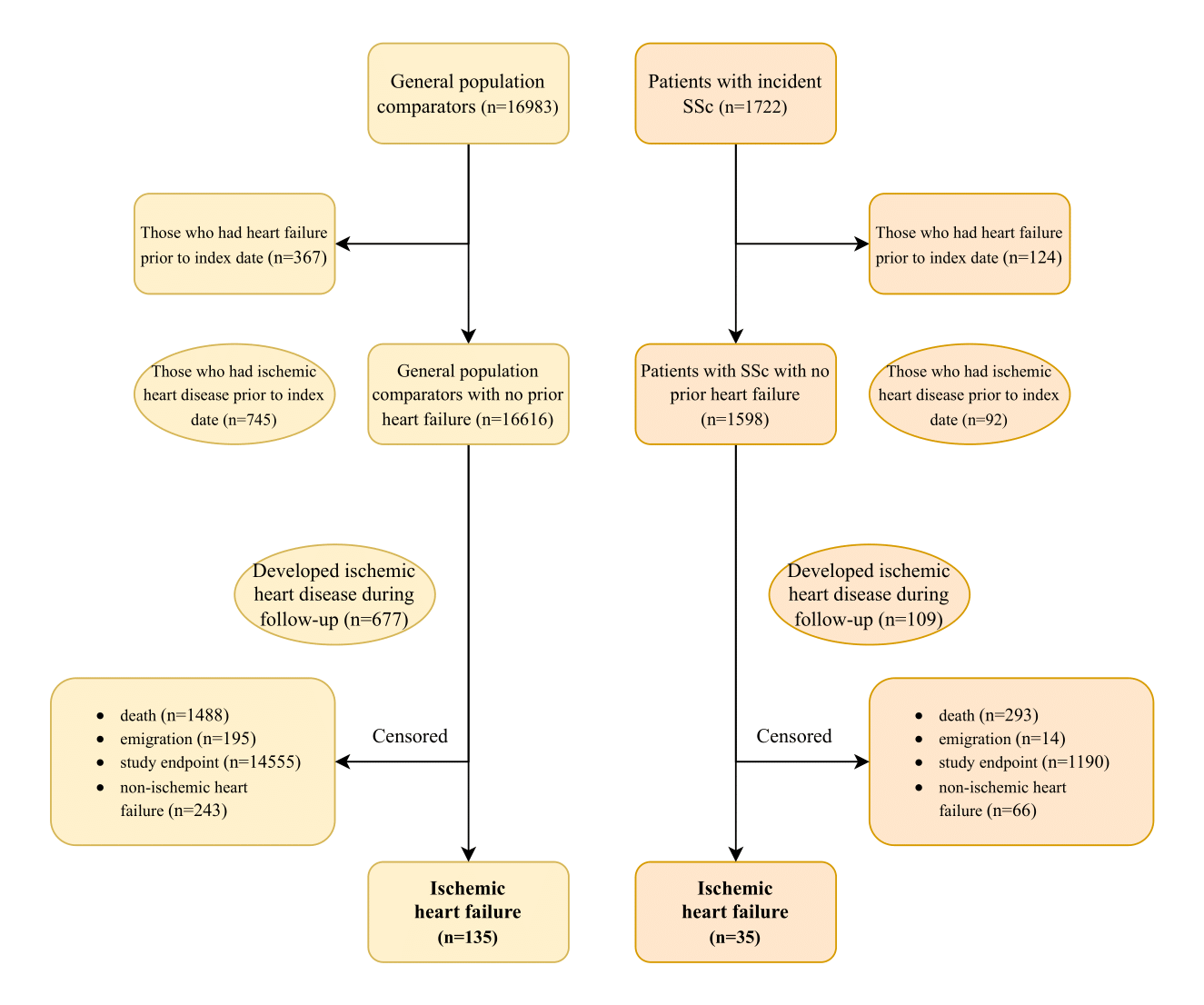


**Supplementary figure S4.** Flowchart of ischemic heart failure.


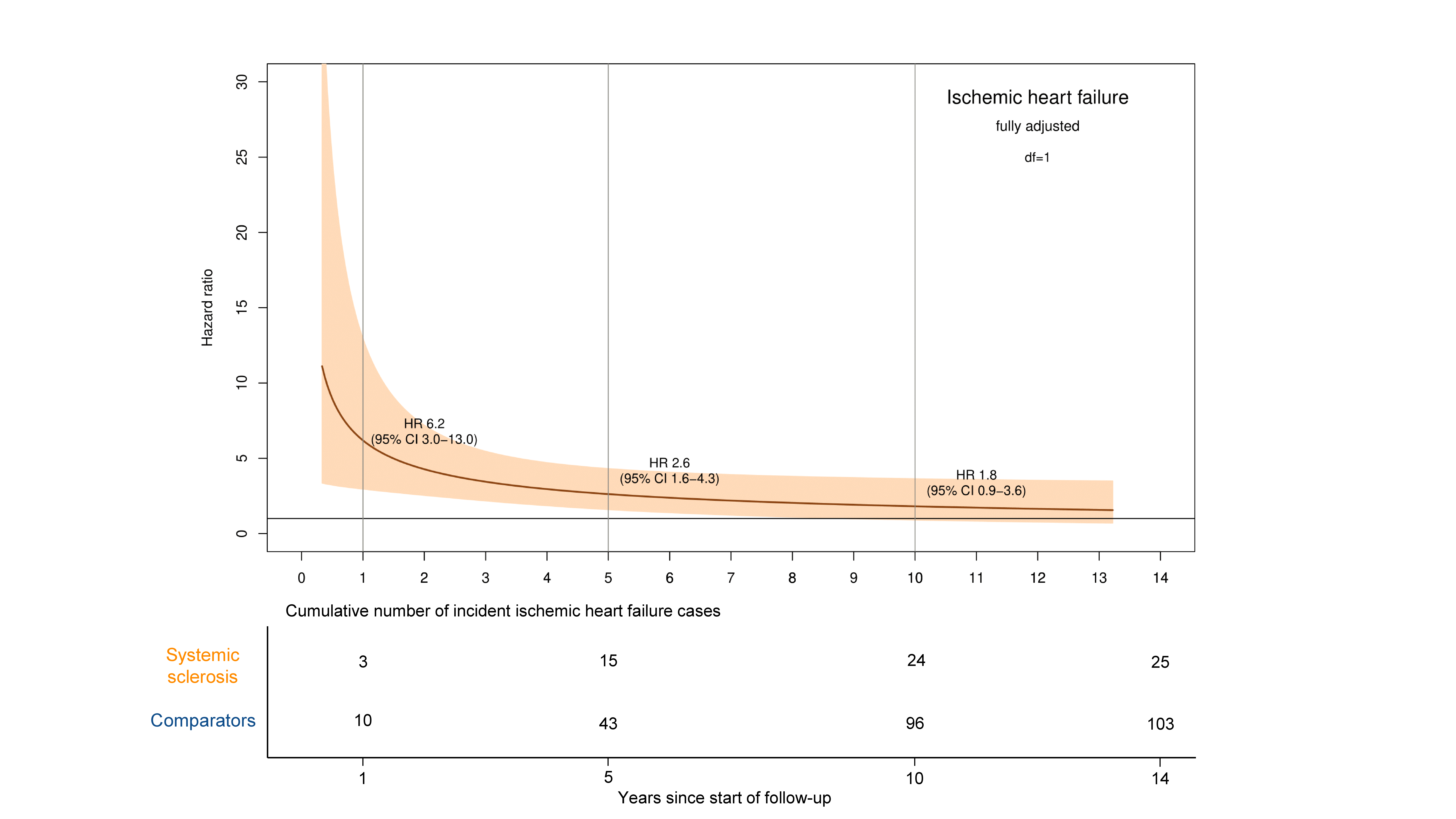


**Supplementary figure S5.** Hazard ratio of ischemic heart failure in patients with systemic sclerosis compared to the general population comparators with **start of follow-up from January 1, 2006 (1,421 patients with systemic sclerosis and 14,809 matched comparators)** using flexible parametric models adjusted for age, sex, education level, ischemic heart disease, atrial fibrillation and flutter, renal diseases, asthma/chronic obstructive pulmonary disease, diabetes mellitus, hyperlipidaemia, ischemic stroke, peripheral artery disease, and hypertension, allowing for systemic sclerosis to have a time-dependent effect.


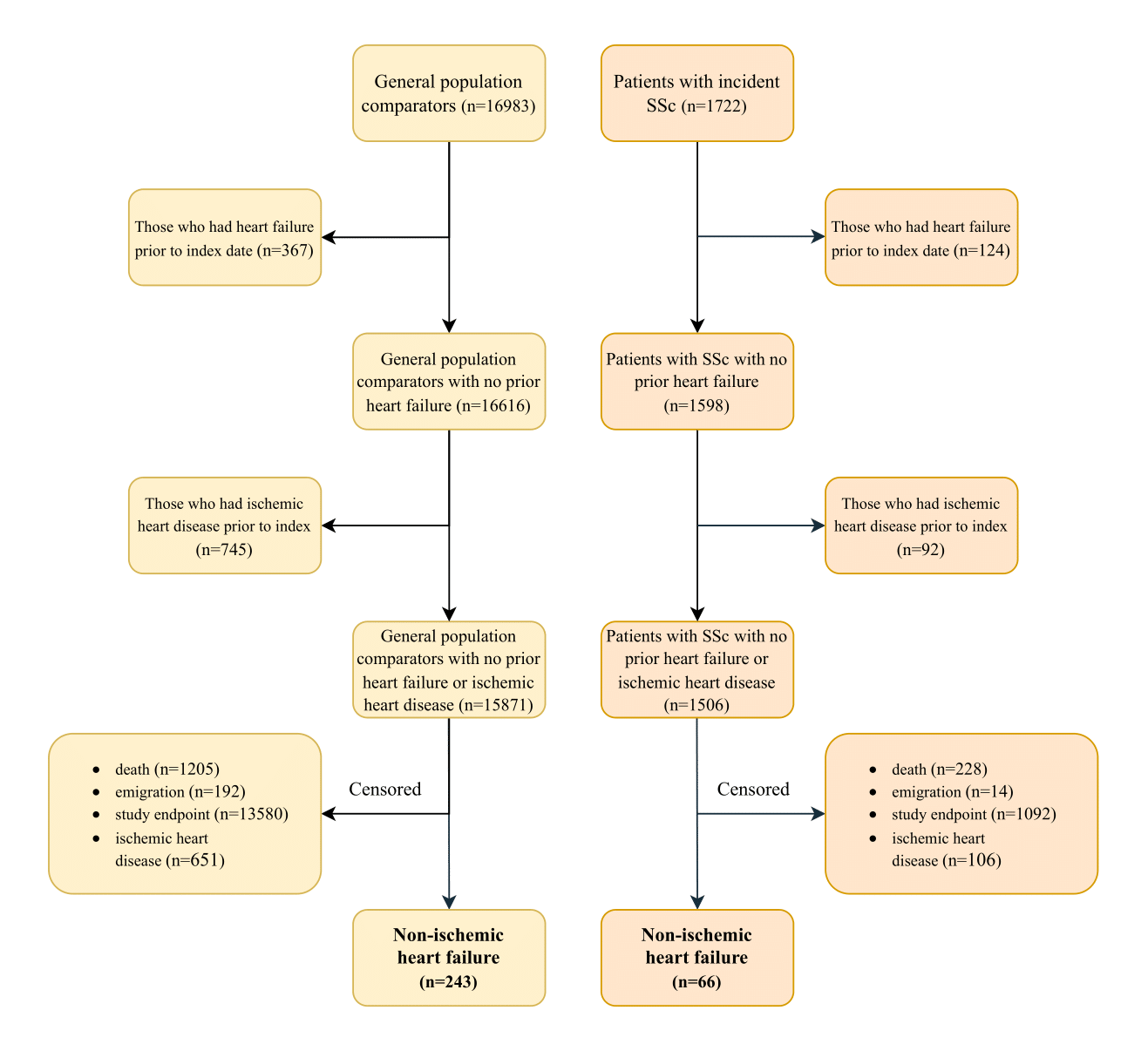


**Supplementary figure S6.** Flowchart of non-ischemic heart failure.


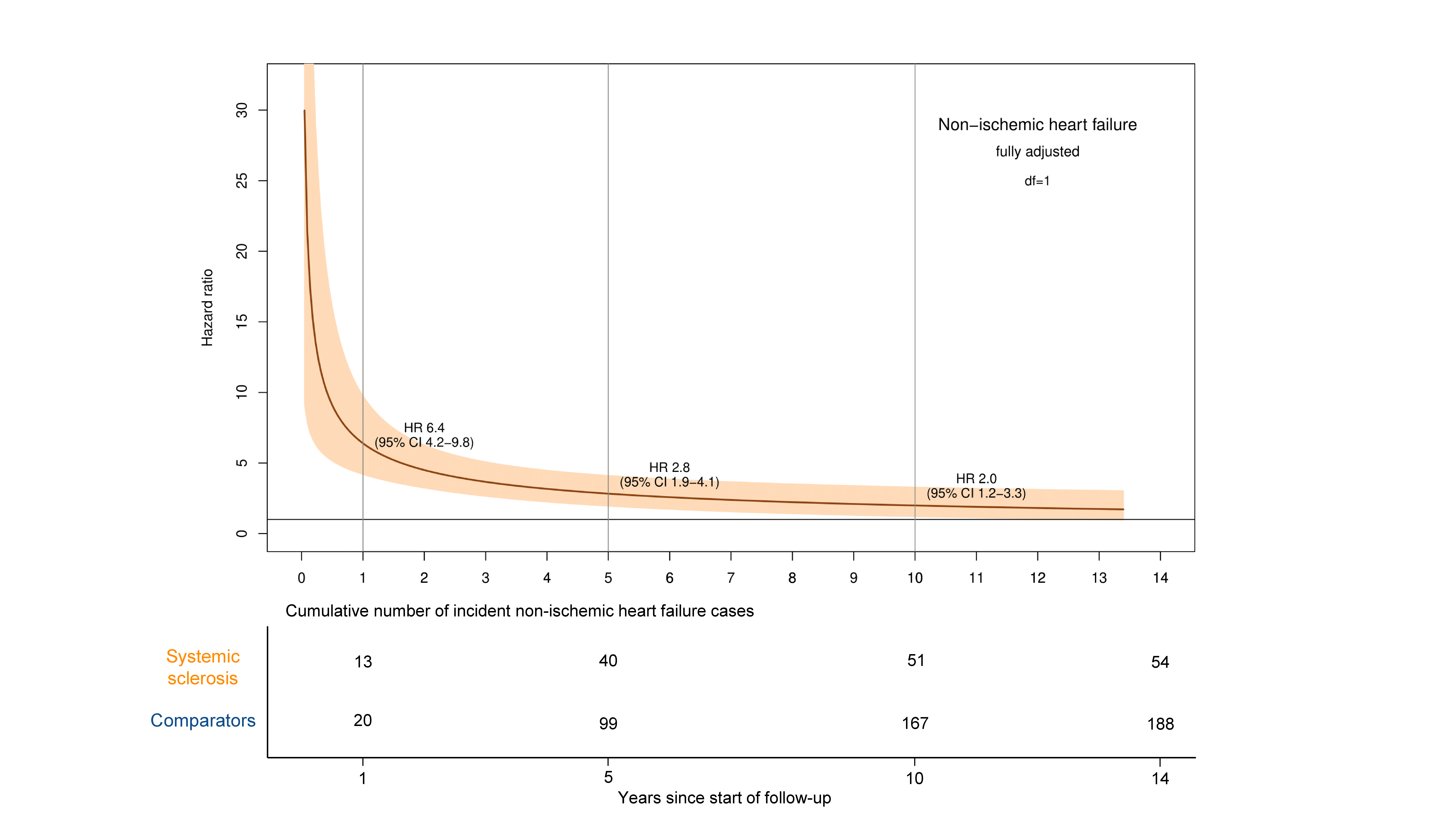


**Supplementary figure S7.** Hazard ratio of non-ischemic heart failure in patients with systemic sclerosis compared to the general population comparators with **start of follow-up from January 1, 2006 (1,335 patients with systemic sclerosis and 14,126 matched comparators)** using flexible parametric models adjusted for age, sex, education level, atrial fibrillation and flutter, renal diseases, asthma/chronic obstructive pulmonary disease, diabetes mellitus, hyperlipidaemia, ischemic stroke, peripheral artery disease, and hypertension, allowing for systemic sclerosis to have a time-dependent effect.

**Supplementary table S1**. Crude incidence rate and rate difference of heart failure overall in patients with systemic sclerosis and general population comparators in several analyses. *IRs are per 10,000 person-years.*

|  | Patients with systemic sclerosis | | | General population Comparators | | |  |
| --- | --- | --- | --- | --- | --- | --- | --- |
|  | **N/total** | **Person-years** | **IR with 95% CI** | **N/total** | **Person-years** | **IR with 95% CI** | **Rate difference with 95% CI** |
| Starting follow-up 90 days after the second visit indicating systemic sclerosis | | | | | | | |
|  | 97/1,559 | 9,390 | 103.3 (83.8-126.0) | 374/16,338 | 110,694 | 33.8 (30.4-37.4) | 69.5 (48.7-90.4) |
| Heart failure redefined as main or contributory diagnosis | | | | | | | |
|  | 175/1,598 | 9,614 | 182.0 (156.1-211.1) | 663/16,616 | 113,974 | 58.2 (53.8-62.8) | 123.9 (96.5-151.2) |
| Excluding individuals with cardiomyopathy prior to start of follow-up | | | | | | | |
|  | 100/1,593 | 9,764 | 102.4 (83.3-124.6) | 372/16,592 | 114,621 | 32.5 (29.2-35.9) | 70.0 (49.6-90.3) |
| Individuals with no history of cardiovascular comorbidities* | | | | | | | |
|  | 23/620 | 3,842 | 59.9 (38.0-89.8) | 64/8050 | 53,696 | 11.9 (9.2-15.2) | 47.9 (23.3-72.6) |

* Individuals with **start of follow-up from January 1, 2006 with** no history of ischemic heart disease, atrial fibrillation and flutter, renal diseases, asthma/chronic obstructive pulmonary disease, diabetes mellitus, hyperlipidaemia, ischemic stroke, peripheral artery disease, and hypertension.


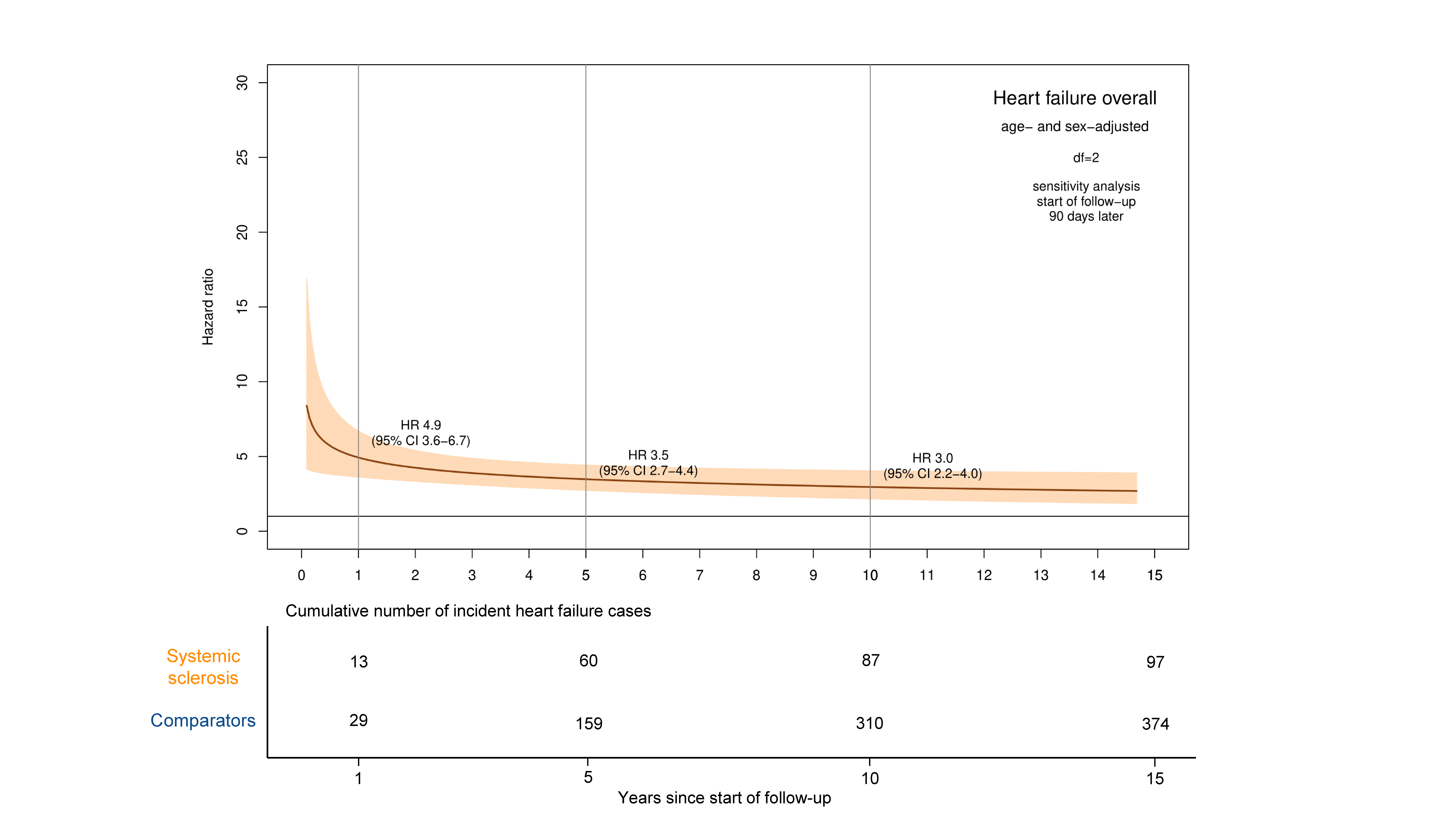


**Supplementary figure S8.** Hazard ratio of heart failure overall in patients with systemic sclerosis compared to the general population comparators **(1,559 patients with systemic sclerosis and 16,338 matched comparators)** starting follow-up 90 days after start of follow-up in the main analysis (the second visit indicating systemic sclerosis), using flexible parametric models adjusted for age and sex, allowing for systemic sclerosis to have a time-dependent effect.


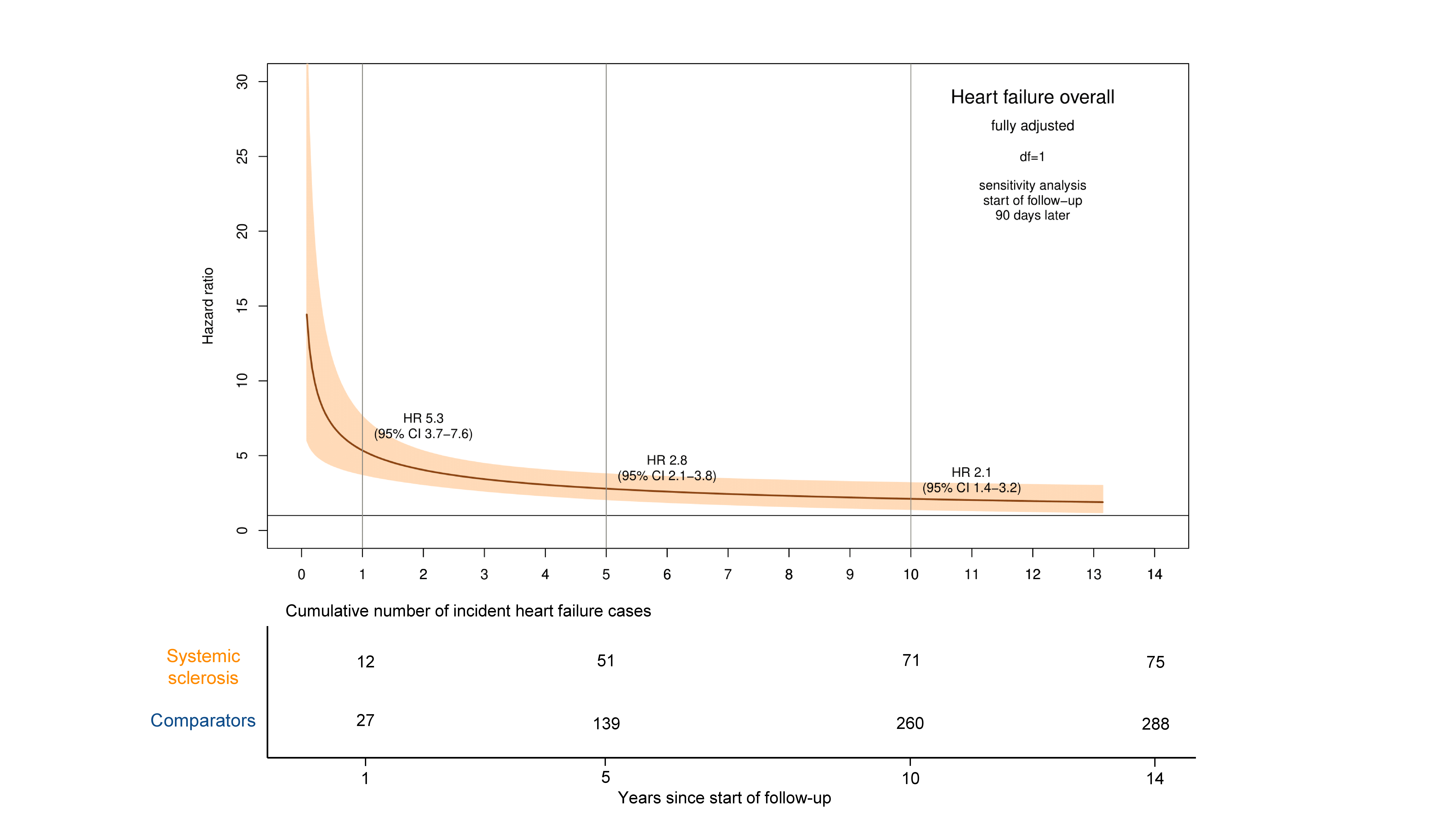


**Supplementary figure S9.** Hazard ratio of heart failure overall in patients with systemic sclerosis compared to the general population comparators with **start of follow-up from January 1, 2006 (1,385 patients with systemic sclerosis and 14,539 matched comparators)** starting follow-up 90 days after start of follow-up in the main analysis (the second visit indicating systemic sclerosis), using flexible parametric models adjusted for age, sex, education level, ischemic heart disease, atrial fibrillation and flutter, renal diseases, asthma/chronic obstructive pulmonary disease, diabetes mellitus, hyperlipidaemia, ischemic stroke, peripheral artery disease, and hypertension, allowing for systemic sclerosis to have a time-dependent effect.


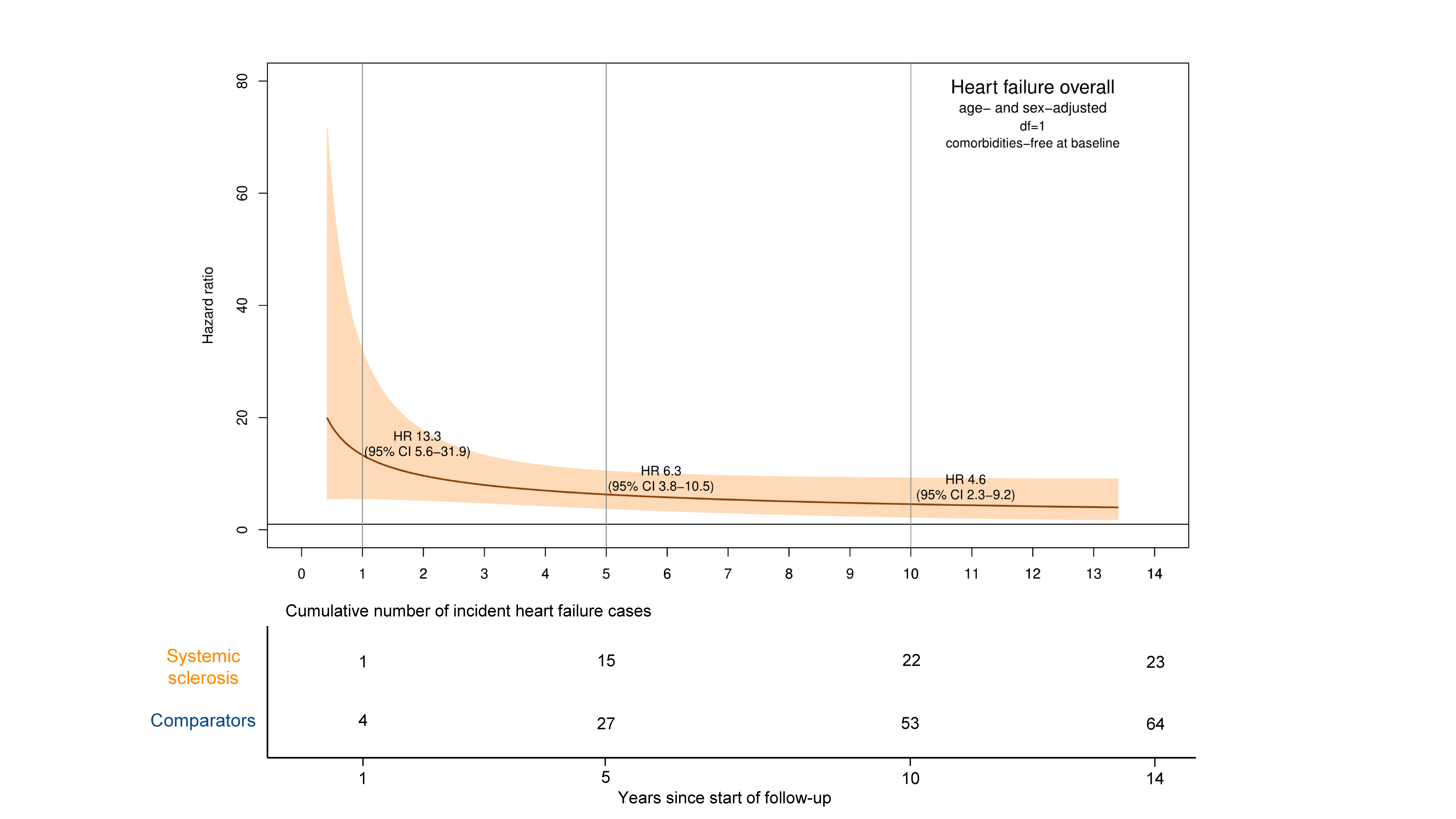


**Supplementary figure S10**. Hazard ratio of heart failure overall in patients with systemic sclerosis compared to the general population comparators with **start of follow-up from January 1, 2006 (620 patients and 8,050 matched comparators)** in those with no history of ischemic heart disease, atrial fibrillation and flutter, renal diseases, asthma/chronic obstructive pulmonary disease, diabetes mellitus, hyperlipidaemia, ischemic stroke, peripheral artery disease, and hypertension, using flexible parametric models adjusted for age and sex, allowing for systemic sclerosis to have a time-dependent effect.


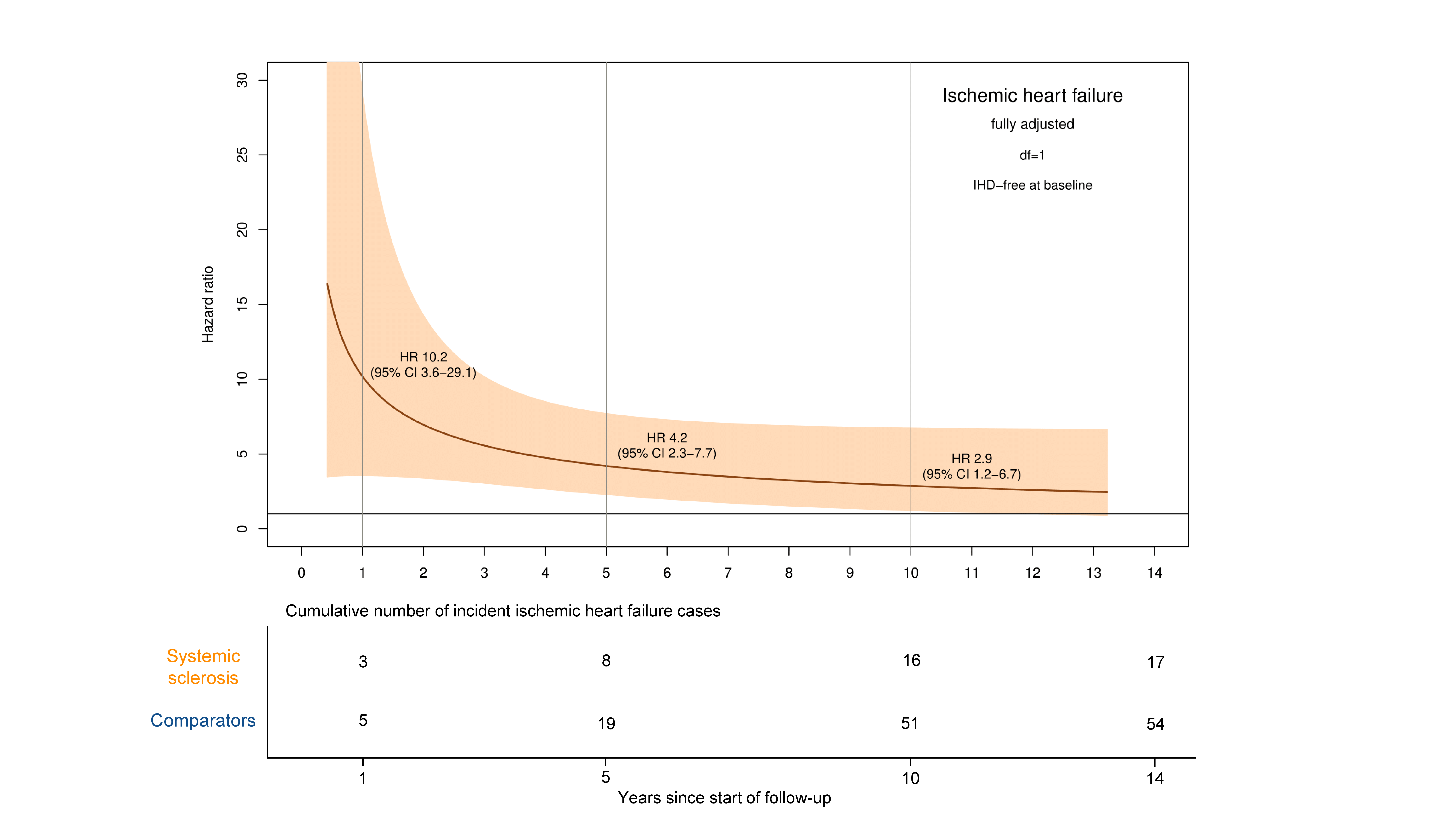


**Supplementary figure S11.** Hazard ratio of ischemic heart failure in patients with systemic sclerosis compared to the general population comparators with **start of follow-up from January 1, 2006 (1,335 patients and 14,126 matched comparators)** in those with no history of ischemic heart disease using flexible parametric models adjusted for age, sex, education level, atrial fibrillation and flutter, renal diseases, asthma/chronic obstructive pulmonary disease, diabetes mellitus, hyperlipidaemia, ischemic stroke, peripheral artery disease, and hypertension, allowing for systemic sclerosis to have a time-dependent effect.

**Supplementary table S2**. Sensitivity analysis. Crude incidence rate of ischemic heart failure (defined as heart failure with ischemic heart disease **up to 30 days** after heart failure diagnosis) and non-ischemic heart failure (defined as heart failure with no ischemic heart disease **up to 30 days** after heart failure diagnosis). *IRs are per 10,000 person-years.*

|  | Patients with systemic sclerosis | | | General population Comparators | | |  |
| --- | --- | --- | --- | --- | --- | --- | --- |
|  | **N** | **Person-years** | **IR with 95% CI** | **N** | **Person-years** | **IR with 95% CI** | **Rate difference with 95% CI** |
| Ischemic heart failure | 36 | 9,779 | 36.8 (25.8-51.0) | 136 | 114,759 | 11.9 (9.9-14.0) | 25.0 (12.8-37.1) |
| Non-ischemic heart failure | 65 | 8,974 | 72.4 (55.9-92.3) | 242 | 107,671 | 22.5 (19.7-25.5) | 50.0 (32.1-67.8) |

**References**

1. Hammar N, Alfredsson L, Rosen M, Spetz CL, Kahan T, Ysberg AS. A national record linkage to study acute myocardial infarction incidence and case fatality in Sweden. Int J Epidemiol. 2001;30 Suppl 1:S30-4.

2. Haupt D, Wettermark B, Nilsson JL. Dispensed volumes of anti-asthmatic drugs related to the prevalence of asthma and COPD in Sweden. Pharmacoepidemiol Drug Saf. 2008;17(5):461-7.

3. Andersson T, Ahlbom A, Carlsson S. Diabetes Prevalence in Sweden at Present and Projections for Year 2050. PLoS One. 2015;10(11):e0143084.

4. Hvidberg MF, Johnsen SP, Glumer C, Petersen KD, Olesen AV, Ehlers L. Catalog of 199 register-based definitions of chronic conditions. Scand J Public Health. 2016;44(5):462-79.
